# Evolution of inspiratory muscle function in children during mechanical ventilation

**DOI:** 10.1186/s13054-021-03647-w

**Published:** 2021-06-30

**Authors:** Benjamin Crulli, Atsushi Kawaguchi, Jean-Paul Praud, Basil J. Petrof, Karen Harrington, Guillaume Emeriaud

**Affiliations:** 1 Pediatric Intensive Care Unit, CHU Sainte-Justine, Université de Montréal, 3175 chemin de la Côte-Sainte-Catherine, Montreal, QC H3T 1C5 Canada; 2 Pediatric Intensive Care Unit, Children’s Hospital of Eastern Ontario, University of Ottawa, 401 Smyth Road, Ottawa, ON K1H 8L1 Canada; 3 Department of Intensive Care Medicine, Tokyo Women’s Medical University, Tokyo, Japan; 4 Neonatal Respiratory Research Unit, Departments of Pediatrics and Pharmacology-Physiology, Université de Sherbrooke, 3001 12e Avenue Nord, Sherbrooke, QC J1H 5N4 Canada; 5 Meakins-Christie Laboratories and Translational Research in Respiratory Diseases Program, McGill University Health Centre and Research Institute, 1001 Boulevard Décarie, Montreal, QC H4A 3J1 Canada

**Keywords:** Mechanical ventilation, Diaphragm, Inspiratory muscle dysfunction, Electrical activity of the diaphragm, Children, Intensive care

## Abstract

**Background:**

There is no universally accepted method to assess the pressure-generating capacity of inspiratory muscles in children on mechanical ventilation (MV), and no study describing its evolution over time in this population.

**Methods:**

In this prospective observational study, we have assessed the function of the inspiratory muscles in children on various modes of MV. During brief airway occlusion maneuvers, we simultaneously recorded airway pressure depression at the endotracheal tube (ΔPaw, force generation) and electrical activity of the diaphragm (EAdi, central respiratory drive) over five consecutive inspiratory efforts. The neuro-mechanical efficiency ratio (NME, ΔPaw/EAdi_max_) was also computed. The evolution over time of these indices in a group of children in the pediatric intensive care unit (PICU) was primarily described. As a secondary objective, we compared these values to those measured in a group of children in the operating room (OR).

**Results:**

In the PICU group, although median NME_occl_ decreased over time during MV (regression coefficient − 0.016, *p* = 0.03), maximum ΔPaw_max_ remained unchanged (regression coefficient 0.109, *p* = 0.50). Median NME_occl_ at the first measurement in the PICU group (after 21 h of MV) was significantly lower than at the only measurement in the OR group (1.8 cmH_2_O/µV, *Q*_1_–*Q*_3_ 1.3–2.4 vs. 3.7 cmH_2_O/µV, *Q*_1_–*Q*_3_ 3.5–4.2; *p* = 0.015). Maximum ΔPaw_max_ in the PICU group was, however, not significantly different from the OR group (35.1 cmH_2_O, *Q*_1_–*Q*_3_ 21–58 vs. 31.3 cmH_2_O, *Q*_1_–*Q*_3_ 28.5–35.5; *p* = 0.982).

**Conclusions:**

The function of inspiratory muscles can be monitored at the bedside of children on MV using brief airway occlusions. Inspiratory muscle efficiency was significantly lower in critically ill children than in children undergoing elective surgery, and it decreased over time during MV in critically ill children. This suggests that both critical illness and MV may have an impact on inspiratory muscle efficiency.

## Background

Critically ill children frequently require support with mechanical ventilation (MV) which allows for an improvement in gas exchange and a decrease in work of breathing. It is, however, imperative to limit its duration because of associated severe complications which can increase the duration of MV, length of intensive care unit stay, and therefore costs. In children, these complications classically include nosocomial infections, tracheal injury, lung injury, hemodynamic effects of positive intrathoracic pressures, impact of sedation, etc. [1]. The impact of MV on the function of respiratory muscles, previously overlooked, is now a growing concern. In ICU patients, the function of the diaphragm, as with other striated muscles, can be adversely affected by critical illness and therapies (ICU-acquired diaphragm dysfunction, ICU–DD) [2], and also more specifically by MV itself (ventilator-induced diaphragm dysfunction, VIDD) [3]. Diaphragm injury can result from insufficient respiratory effort secondary to over-assistance by MV [4], from excessive inspiratory effort due to under-assistance leading to fatigue [5], or finally from contractile activation, while the muscle is lengthening (e.g., during asynchrony or hyperinflation) [6]. Studies in both animal models and humans have shown that MV is associated with a series of molecular changes in the diaphragm muscle [7]. A decrease in its force-generating capacity ensues [8–10], which is then followed by the development of micro- [11, 12] and macroscopic atrophy [13–18] in muscle fibers leading to even greater muscle weakness [9, 12, 19].

ICU–DD is described in both early [10] and late [20, 21] phases of critical illness, and its risk factors are numerous. The majority of adult patients present severe diaphragmatic dysfunction on admission to the ICU, before any prolonged duration of MV [10]. VIDD is highly prevalent in adult critical care (63–84% of patients depending on the phase of critical illness) [10, 20–23] and is associated with worse outcomes such as ventilation weaning failure [20, 21, 24, 25], longer duration of MV [8, 23, 24, 26, 27], prolonged ICU admission [27], and increased mortality [21, 23, 26, 28].

Interestingly, respiratory physiology in children has some specific features which may render the diaphragm particularly susceptible to dysfunction. Because of a more compliant abdomen and a smaller area of apposition between the diaphragm and the rib cage, contraction of the diaphragm in infants does not result in the same degree of expansion of the lower ribs as in adults [29]. In addition, the highly compliant chest wall [30] and horizontal insertion of the ribs can result in chest wall distortion during inspiration in the context of rapid eye movement sleep [31] or respiratory distress. Combined with small distal airways, this relatively high parietal compliance in neonates and infants requires that end-expiratory lung volume be maintained above the relaxation volume of the respiratory system [32], referred to as dynamic hyperinflation. This is achieved by expiratory airflow retardation resulting from the contraction of laryngeal muscles (in children without an artificial airway) or by persistent activation of the diaphragm during expiration [32]. The diaphragm may therefore be active during both inspiration and expiration [33, 34]. In contrast, the diaphragm is very frequently inactive during pediatric conventional MV [35].

The methods used in the assessment of inspiratory muscle function in children are not well established [36] and—to the best of our knowledge—no study has described the evolution of the efficiency of these muscles in critically ill children on MV.

In the present study, our primary objective was to test the hypothesis that inspiratory muscle efficiency decreases over time in mechanically ventilated critically ill children during the first 3 days of invasive ventilation. We first developed a standardized method to measure inspiratory muscle function in this setting (objective 1a) using the airway pressure at the endotracheal tube (Paw), the electrical activity of the diaphragm (EAdi), and the Paw/EAdi ratio which provides an estimate of the ability to generate pressure normalized to neural drive (neuro-mechanical efficiency, NME) [37]. We subsequently determined the evolution of Paw and NME over time in critically ill children on MV in the PICU (objective 1b). As a secondary objective, we compared these indices of inspiratory muscle function in this population to a group of healthy children briefly ventilated during general anesthesia for elective surgery (objective 2).

## Methods

This was a prospective single-center observational study performed in a tertiary academic center, in both the pediatric intensive care unit (PICU) and the operating room (OR). Approval by the Ethics Committee of the CHU Sainte-Justine Research Center was granted before initiating enrollment (2017–1534). Written informed consent was obtained from the parents/guardians by a member of the research team.

### Study population

In the PICU group, inclusion criteria consisted of children (defined as age between 1 week and 18 years) on invasive ventilation for less than 24 h and for which extubation was not planned in the following 24 h. Inclusion criteria in the OR group consisted of children with no chronic or acute respiratory disease undergoing endotracheal intubation for elective otorhinolaryngological surgery without planned use of neuromuscular blockade. In both groups, we excluded pre-existing conditions with a possible impact on inspiratory muscle function (global neuromuscular disease, cervical spine injury, bi-hemispheric or brain stem lesions, known diaphragmatic disease, uni- or bi-lateral phrenic paralysis, recent thoracic surgery, recent multiple rib fractures), patients in whom the placement of a nasogastric tube was contraindicated (trauma or recent surgery in cervical or nasopharyngeal regions, severe coagulation disorder), in whom muscle function was artificially suppressed (use of neuromuscular blockade in the 2 h prior to inclusion), or in whom life-sustaining treatment was being withheld.

### Study protocol

**Fig. 1:**
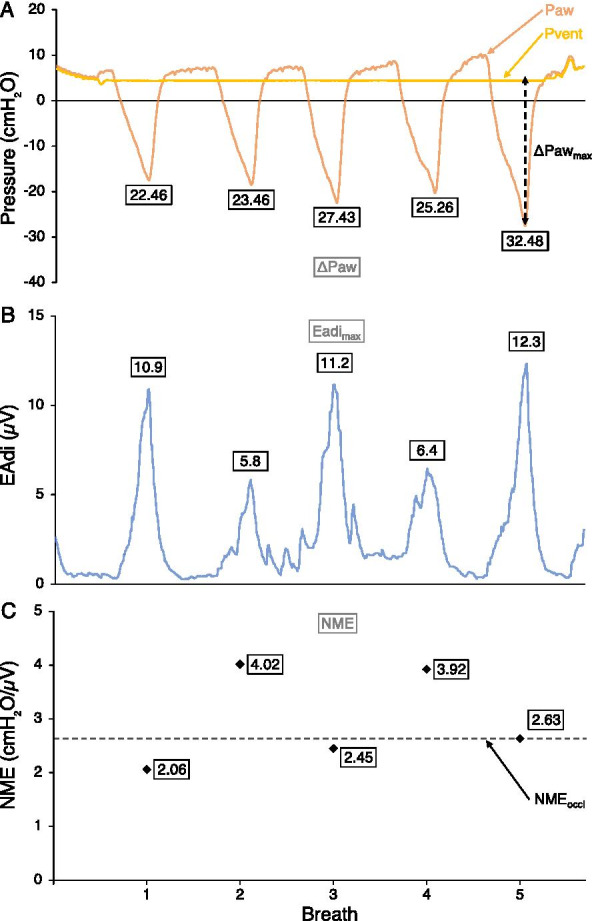
Example of recorded signals and calculated variables during an occlusion maneuver. Paw denotes airway pressure, Pvent pressure on ventilator circuit, EAdi electrical activity of the diaphragm, NME neuro-mechanical efficiency ratio (defined as ΔPaw divided by EAdi_max_). **A** Paw and Pvent over time, with ΔPaw values specified for each breath, dotted arrow shows ΔPaw_max_. **B** EAdi over time, with EAdi_max_ values specified for each breath. **C** NME values for each breath, with the median of all 5 values (NME_occl_) as a dotted line

After enrollment, a dedicated 6Fr or 8Fr nasogastric catheter (Maquet critical care, Solna, Sweden) was inserted to a depth determined by a validated equation and adjusted using dedicated software on the Servo-i ventilator, which then processed and displayed the EAdi signal [38], as per the manufacturer’s recommendations. Patients were placed in a supine position, and the respiratory circuit was assessed for leaks (all had a cuffed endotracheal tube). If needed, an opioid dose was administered (as prescribed by the treating team) and the endotracheal tube was suctioned. A low dead space (3.2 ml) pneumatic occlusion valve (2820 Series, Hans Rudolph, Southport, UK) was installed between the ventilator circuit and the endotracheal tube. An expiratory occlusion maneuver was performed (at the current level of PEEP in the PICU group, without PEEP in the OR group), and we simultaneously recorded negative Paw generated by the patient and EAdi signal over 5 consecutive breaths (Fig. 1). The maneuvers were subsequently repeated three times with at least a 1-min interval. The first measurement of diaphragm contractile strength was conducted as soon as possible after inclusion, within 24 h of intubation. Measurements were then repeated as close as possible to the following time points after the first measurement: 12 h, 24 h, 48 h and 72 h or until extubation/death. In the OR group, only one measurement was performed immediately after intubation and before surgery. Clinical data were recorded at baseline and at each measurement. Relevant outcome measures including mortality, extubation failure, prolonged (> 72 h) post-extubation non-invasive ventilation (NIV), and PICU length of stay were later assessed at the time of discharge from PICU.

### Data acquisition

EAdi waveforms were acquired from the Servo-i ventilator (Maquet critical care, Solna, Sweden) via a RS232 serial port. Pressure measurements were taken with a manometer connected to the endotracheal tube via the occlusion valve. The signals were simultaneously displayed and recorded using dedicated software (NeuroVent Research, Toronto, Canada). Baseline values of EAdi (maximum EAdi value in each minute) were also extracted from the ventilator for up to 60 min before each measurement in the PICU group. For each breath during each occlusion maneuver, the peak value of EAdi was recorded (EAdi_max_) as well as the difference in pressure between the lowest Paw and the preceding end-expiratory pressure (ΔPaw). In order to compensate for central respiratory drive, a neuro-mechanical efficiency ratio (NME) was computed by dividing ΔPaw by EAdi_max_ for each breath (Fig. 1). The most reliable value of ΔPaw and NME to report for a given occlusion maneuver was determined by assessing, using coefficients of variation (CoV), the within-measurement variability of different metrics over three occlusion maneuvers (first breath, last breath, breath with largest ΔPaw deflection, breath with largest NME value, and median value over all breaths) in the PICU group. A summary of variables used in this article is provided to assist the reader (Table 1).

**Table 1:** Nomenclature of variables used

Paw	Pressure signal recorded at endotracheal tube
ΔPaw	Difference between the lowest Paw during an occluded breath and preceding end-expiratory Paw
ΔPaw_max_	Largest ΔPaw deflection amongst 5 breaths of an occlusion maneuver
Maximum ΔPaw_max_	Largest ΔPaw_max_ deflection over 3 occlusion maneuvers of a single recording in time
ΔPaw_twitch_	Pressure decrease at occluded endotracheal tube generated by magnetic stimulation of the phrenic nerves
EAdi	Electrical signal recorded by electrodes in esophagus
EAdi_max_	Peak value of EAdi during an occluded breath
NME	Ratio of ΔPaw to EAdi_max_ for an occluded breath
NME_occl_	Median NME value amongst 5 breaths of an occlusion maneuver
Median NME_occl_	Median NME_occl_ value over 3 occlusion maneuvers of a single recording in time

### Sample size

At the time of designing the study, no data on inspiratory muscle efficiency in children on MV were available. In adult patients, data on VIDD are scant but Jaber et al. [9] demonstrated a strong and homogenous decrease in the strength of the diaphragm over 5–6 days of MV with only 6 patients. In this context, a sample size of 20 patients was chosen for the PICU group in order to improve external validity. In the OR group, low heterogeneity was expected and a convenient sample of 10 patients was selected.

### Statistical analyses

Data were reported as median (first–third quartiles, *Q*_1_–*Q*_3_), unless stated otherwise. Linear mixed-effects models were then used to explore the effect of time on MV on maximum ΔPaw_max_ and median NME_occl_, using time as variable with a fixed effect, and individual patient as variable with a random effect. Aiming to weigh the impact of critical illness on inspiratory muscle function, independently from MV, a Mann–Whitney U test was run to determine if there were significant differences in maximum ΔPaw_max_ and median NME_occl_ between the first measurement of the PICU group and the only measurement of the OR group. The level of significance for all statistical tests was set at *p* < 0.05. Statistical analysis was performed using SPSS (SPSS Statistics, Version 25. Armonk, NY: IBM Corp.) and Stata (Stata Statistical Software, Release 13. College Station, TX: StataCorp LLC.) software.

## Results

Patients were recruited between October 2017 and September 2019. In the PICU group, 223 consecutive intubated patients were screened, 50 met the inclusion and exclusion criteria, 36 families provided consent, and the research team was available to make measurements in 20 patients. Table 2 describes patient characteristics in the PICU group. One patient had severe meningitis and was excluded from the analysis due to a lack of spontaneous breathing activity at any time point. Patients were on invasive ventilation for 63.9 h (*Q*_1_–*Q*_3_ 50.4–77.4) and stayed in the PICU for 5.3 days (*Q*_1_–*Q*_3_ 4–8.8). In the OR group, 12 consecutive eligible patients were approached, 10 families provided consent, and measurements were made in all 10 patients. Because of difficulties in promptly achieving appropriate nasogastric tube positioning between induction of anesthesia and surgery, EAdi signal could not be appropriately recorded for 3 patients in the OR group and NME was therefore not computed. Table 3 describes patient characteristics in the OR group. In total, 59 measurements were made, 147 occlusions performed and 625 breaths analyzed in the PICU group; 10 measurements were made, 30 occlusions performed and 150 breaths analyzed in the OR group.

**Table 2:** Patient characteristics (PICU group, 19 patients)

Age (months), median (*Q*_1_–*Q*_3_)	13.7 (1.5–34)
Weight (kg), median (*Q*_1_–*Q*_3_)	11 (3.8–17.3)
Sex, male (%)	12 (63.2%)
Comorbidity, *n* (%)	
Prematurity	6 (31.6%)
Previous episode of invasive ventilation (within 7 days of intubation)	3 (15.8%)
Reason for PICU admission, *n* (%)	
Neurological (traumatic brain injury)	1 (5.3%)
Neurological (excluding traumatic brain injury)	4 (21.1%)
Respiratory-upper airway	4 (21.1%)
Respiratory-lower airway/pulmonary	7 (36.8%)
Sepsis/shock	1 (5.3%)
Other	2 (10.5%)
Reason for intubation, *n* (%)	
Neurological	4 (21.1%)
Apnea	3 (15.8%)
Respiratory-upper airway	4 (21.1%)
Respiratory-lower airway/pulmonary	6 (31.6%)
Hemodynamics	1 (5.3%)
Procedure	1 (5.3%)
Drugs used during study period, *n* (%)	
Paralysis (excluding for intubation)	5 (26.3%)
Corticosteroids	8 (42.1%)
Inotropes/vasodilators	5 (26.3%)
Spontaneous mode used during MV episode (NAVA/PSV), *n* (%)	14 (73.7%)
Number of measurements, median (*Q*_1_–*Q*_3_)	3 (2.5–4)
PICU outcomes	
Duration of MV (hours), median (*Q*_1_–*Q*_3_)	63.9 (50.4–77.4)
Reintubation within 24 h of extubation, *n* (%)	0 (0%)
Rescue NIV for more than 72 h post-extubation, *n* (%)	2 (10.5%)
PICU length of stay (days), median (*Q*_1_–*Q*_3_)	5.3 (4–8.8)
Died during PICU admission, *n* (%)	0 (0%)

*PICU* pediatric intensive care unit, *Q*_1_–*Q*_3_ first and third quartiles, *NAVA* neurally adjusted ventilatory assist, *PSV* pressure-support ventilation, *MV* mechanical ventilation, *NIV* non-invasive ventilation

**Table 3:** Patient characteristics (OR group, 10 patients)

Age (months), median (*Q*_1_–*Q*_3_)	59.3 (46.6–65.8)
Weight (kg), median (*Q*_1_–*Q*_3_)	17.9 (13.6–23)
Sex, male (%)	5 (50%)
Comorbidity, *n* (%)	
Prematurity	1 (10%)
Pediatric-adapted ASA-PS score, median (*Q*_1_–*Q*_3_)	2.5 (2–3)
Analgesia and sedation received for induction	
Sevoflurane, *n* (%)	10 (100%)
Opioids-fentanyl, *n* (%), median dose (mcg/kg) (*Q*_1_–*Q*_3_)	4 (40%) 1 (0.8–1.1)
Opioids-remifentanil, *n* (%), median dose (mcg/kg) (*Q*_1_–*Q*_3_)	4 (40%) 1.5 (1.5–2.8)^a^
Benzodiazepines, *n* (%)	0 (0%)
Propofol, *n* (%), median dose (mg/kg) (*Q*_1_–*Q*_3_)	10 (100%) 1.9 (1.8–2.5)^a^
Dexmedetomidine, *n* (%), median dose (mcg/kg) (*Q*_1_–*Q*_3_)	4 (40%) 0.5 (0.4–0.6)
Ketamine, *n* (%), dose (mg/kg)	1 (10%) 0.5
Other drugs received before measurement	
Dexamethasone, *n* (%), median dose (mg/kg) (*Q*_1_–*Q*_3_)	10 (100%) 0.1 (0.1–0.1)
Duration of MV on first measurement (minutes), median (*Q*_1_–*Q*_3_)	7.6 (2.6–12.4)
Maximum ΔPaw_max_ on first measurement (cmH_2_O), median (*Q*_1_–*Q*_3_)	31.3 (28.5–35.5)
Median NME_occl_ on first measurement (cmH_2_O/µV), median (*Q*_1_–*Q*_3_)	3.6 (3.5–4.2)
Hospital length of stay (days), median (*Q*_1_–*Q*_3_)	1.5 (1–2.8)

OR denotes operating room, *Q*_1_–*Q*_3_ first and third quartiles, ASA-PS American Society of Anesthesiologists physical status [61], MV mechanical ventilation, ΔPaw_max_ maximum inspiratory airway pressure deflection over 5 occluded breaths, NME_occl_ median neuro-mechanical efficiency ratio (defined as inspiratory airway pressure divided by peak electrical activity of the diaphragm) over 5 occluded breaths

^a^One patient received drugs as continuous infusions and accurate doses could not be reported

Clinical parameters and ventilator settings at each measurement in the PICU group are shown in Table 4. First measurements for each patient were made at 20.7 h (*Q*_1_–*Q*_3_ 13.9–21.7). A median of 39.2 h (*Q*_1_–*Q*_3_ 22.9–44.9) elapsed between the first and the last measurement. The last measurement was obtained at a median of 4.8 h (*Q*_1_–*Q*_3_ 1.5–25) before extubation. Spontaneous modes of ventilation (pressure-support ventilation, PSV or neurally adjusted ventilatory assist, NAVA) were increasingly used in the later measurements. Baseline EAdi in the 60 min before the last measurement was 9.3 µV (*Q*_1_–*Q*_3_ 6.6–13.7) compared to 3.8 µV (*Q*_1_–*Q*_3_ 2.9–7.2) before the first measurement.

**Table 4:** Clinical condition, ventilatory parameters, and recorded values at each measurement (PICU group)

	All	Measurement 1	Measurement 2	Measurement 3	Measurement 4
Number of measurements	59	19	18	14	8
Duration of MV at measurement (hours), median (*Q*_1_–*Q*_3_)	34.7 (22.9–49.6)	20.7 (13.9–21.7)	33.5 (30.4–38.4)	49.6 (43.1–59.7)	69.3 (58.4–80.8)
PELOD-2 score, median (*Q*_1_–*Q*_3_)	8 (6–9)	9 (7.5–11.5)	7 (5.3–8.8)	7 (5.3–8)	8 (6.5–8.3)
Sedation used within 4 h before measurements, *n* (%)					
Opioids	52 (88.1%)	18 (94.7%)	17 (94.4%)	11 (78.6%)	6 (75%)
Benzodiazepines	17 (28.8%)	5 (26.3%)	5 (27.8%)	5 (35.7%)	2 (25%)
Propofol	1 (1.7%)	0 (0%)	0 (0%)	0 (0%)	1 (12.5%)
Dexmedetomidine	32 (54.2%)	11 (57.9%)	11 (61.1%)	9 (64.3%)	1 (12.5%)
Total opioid received before measurements, *n* (%), median morphine equivalent dose (mg/kg) (*Q*_1_–*Q*_3_)					
Within 4 h before measurements	52 (88.1%) 0.28 (0.15–0.53)	18 (94.7%) 0.45 (0.15–0.54)	17 (94.4%) 0.4 (0.17–0.53)	11 (78.6%) 0.23 (0.16–0.34)	6 (75%) 0.24 (0.18–0.34)
Within 30 min before measurements	38 (64.4%) 0.08 (0.04–0.14)	13 (68.4%) 0.08 (0.05–0.14)	13 (72.2%) 0.08 (0.05–0.14)	7 (50%) 0.03 (0.02–0.07)	5 (62.5%) 0.12 (0.08–0.19)
Sedation scores at measurements, median (*Q*_1_–*Q*_3_)					
RASS	− 1 (− 2 to 1)	− 1 (− 3 to 0.5)	− 1 (− 1.5 to − 1)	− 1 (− 1.5 to − 0.8)	2 (2–2)
COMFORT-B	12 (10–14)	12 (9–13)	12 (9.8–13)	12 (11–13.8)	14 (12–17.3)
Ventilation mode, *n* (%)					
Assist, volume-control	6 (10.2%)	3 (15.8%)	3 (16.7%)	0 (0%)	0 (0%)
Assist, pressure-control	13 (22%)	5 (26.3%)	4 (22.2%)	3 (21.4%)	1 (12.5%)
Assist, pressure-regulated volume control	13 (22%)	6 (31.6%)	4 (22.2%)	1 (7.1%)	2 (25%)
Support, pressure-control (PSV)	15 (25.4%)	4 (21.1%)	4 (22.2%)	5 (35.7%)	2 (25%)
Support, neurally adjusted (NAVA)	12 (20.3%)	1 (5.3%)	3 (16.7%)	5 (35.7%)	3 (37.5%)
Ventilation parameters					
Tidal volume (ml/kg), median (*Q*_1_–*Q*_3_)	6.5 (5.2–7.8)	6.5 (5.4–7.4)	6.9 (6.2–8.6)	6.6 (5.2–8.1)	5.7 (5–6.6)
Respiratory rate above set, for non-spontaneous modes (%), median (*Q*_1_–*Q*_3_)	12.4% (3.3–28.9%)	8.7% (1.4–27.9%)	13.3% (7.3–25.7%)	2.2% (0–33.3%)	24% (19.1–93.3%)
NAVA level (cmH_2_O/µV), median (*Q*_1_–*Q*_3_)	0.6 (0.5–1)	0.5	0.5 (0.5–1)	0.8 (0.5–1)	0.6 (0.6–0.8)
Delta pressure (cmH_2_O), median (*Q*_1_–*Q*_3_)	11 (6–14.5)	12 (8.5–18)	11 (8.5–14.8)	7.5 (5.3–12)	5.5 (3.8–12)
PEEP (cmH_2_O), median (*Q*_1_–*Q*_3_)	6 (5–8)	6 (5–8.5)	7 (5–8)	6.5 (5.3–7)	6 (5.8–6.3)
FiO_2_ (%), median (*Q*_1_–*Q*_3_)	35% (25–45%)	40% (25–55%)	32.5% (22–49%)	30.5% (25–40%)	35.5% (30–43%)
Ventilation effectiveness					
SpO_2_ (%), median (*Q*_1_–*Q*_3_)	99% (96–100%)	98% (96–100%)	100% (99–100%)	96.5% (96–99%)	99% (97–100%)
pH, median (*Q*_1_–*Q*_3_)	7.39 (7.35–7.42)	7.4 (7.36–7.43)	7.37 (7.34–7.43)	7.39 (7.35–7.41)	7.39 (7.37–7.42)
PCO_2_ (mmHg), median (*Q*_1_–*Q*_3_)	43.9 (37.8–52.5)	42.1 (35.7–50.1)	43.1 (37.6–50.2)	44.9 (40.9–54.9)	47.3 (43.1–55.4)
Median baseline EAdi_max_ 60 min before measurement (µV), median (*Q*_1_–*Q*_3_)	5.7 (2.9–10)	3.8 (2.9–7.2)	6.5 (2.7–10.5)	5.3 (2.9–11.9)	9.3 (6.6–13.7)
Maximum ΔPaw_max_ (cmH_2_O), median (*Q*_1_–*Q*_3_)	28.7 (20.8–55.4)	35.1 (21–58)	26.7 (21.1–43.1)	27.8 (20.9–41.1)	47.2 (22.2–73.1)
Median NME_occl_ (cmH_2_O/µV), median (*Q*_1_–*Q*_3_)	1.6 (0.9–2.6)	1.8 (1.3–2.4)	1.9 (1.4–2.6)	1.3 (0.9–2.7)	0.9 (0.7–1.6)

PICU denotes pediatric intensive care unit, *Q*_1_–*Q*_3_ first and third quartiles, MV mechanical ventilation, PELOD pediatric logistic organ dysfunction, RASS Richmond agitation–sedation scale, PSV pressure-support ventilation, NAVA neurally adjusted ventilatory assist, PEEP positive end-expiratory pressure, FiO_2_ fractional concentration of inspired oxygen, SpO_2_ oxygen saturation, PCO_2_ blood carbon dioxide pressure, EAdi_max_ peak electrical activity of the diaphragm, ΔPaw_max_ maximum inspiratory airway pressure deflection over 5 occluded breaths, NME_occl_ median neuro-mechanical efficiency ratio (defined as inspiratory airway pressure divided by peak electrical activity of the diaphragm) over 5 occluded breaths

### Assessment of inspiratory muscle function (objective 1a)

**Fig. 2:**
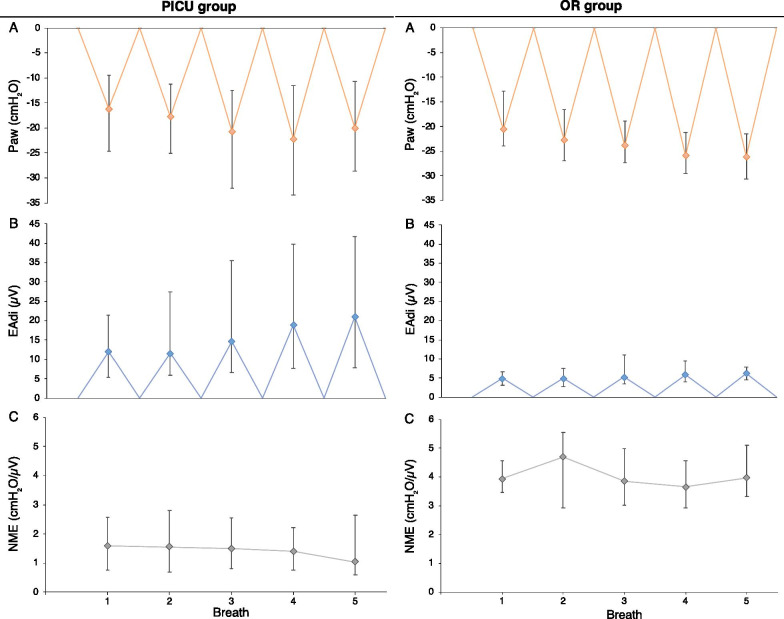
Median evolution of ΔPaw, Eadi_max_ and NME over the 5 occluded breaths in both groups. Data represented as median (dots) and first–third quartiles (whiskers). **A** ΔPaw. **B** EAdi_max_. **C** NME. Left: pediatric intensive care unit (PICU) group, right: operating room (OR) group

To synthesize the evolution of ΔPaw, EAdi_max_ and NME over 5 occluded breaths, medians of all recordings are represented in Fig. 2 for both groups. ΔPaw tends to increase progressively over each breath of the occlusion maneuver (Fig. 2A). This is matched by an increase in respiratory drive, EAdi_max_ (Fig. 2B). The resulting NME value is therefore relatively stable (Fig. 2C, right). Of note, on the last breath, the increase in EAdi_max_ was not paralleled by an increase in ΔPaw, resulting in a decrease in NME (Fig. 2C, left). The variability of the different metrics assessing ΔPaw and NME for each occlusion maneuver in the PICU group is shown in Table 5. For ΔPaw_,_ the value corresponding to the breath with the largest maximum inspiratory airway pressure deflection (ΔPaw_max_) exhibited the lowest variability (CoV of 26.1%) and was used to represent ΔPaw for a single occlusion maneuver. The maximum value of the three occlusion maneuvers was then used to represent ΔPaw_max_ for a given recording in time. For NME, the median value of the 5 breaths in each occlusion maneuver (NME_occl_) exhibited the lowest variability (CoV of 23.7%) and was used to represent NME for a single occlusion maneuver. The median value of the three occlusion maneuvers was then used to represent NME_occl_ for a given recording in time. To assess the impact of the number of breaths on median NME_occl_, we repeated the calculation using only the first 4 breaths and obtained values that were very closely correlated to those calculated on 5 breaths (*R*^2^ 0.98).

**Table 5:** Variability of different metrics of ΔPaw and NME in the PICU group

Mean coefficient of variation					
ΔPaw			NME		
1	Maximum ΔPaw	26.1%	1	Median NME	23.7%
2	Median ΔPaw	26.3%	2	Maximum ΔPaw	26.8%
3	Last breath	29.8%	3	Maximum NME	29.8%
4	Maximum NME	30.3%	4	Last breath	33.8%
5	First breath	35.5%	5	First breath	36.8%

PICU denotes pediatric intensive care unit, ΔPaw maximum inspiratory airway pressure deflection, NME neuro-mechanical efficiency ratio (defined as inspiratory airway pressure divided by peak electrical activity of the diaphragm)

### Evolution of inspiratory muscle function over time (objective 1b)

**Fig. 3:**
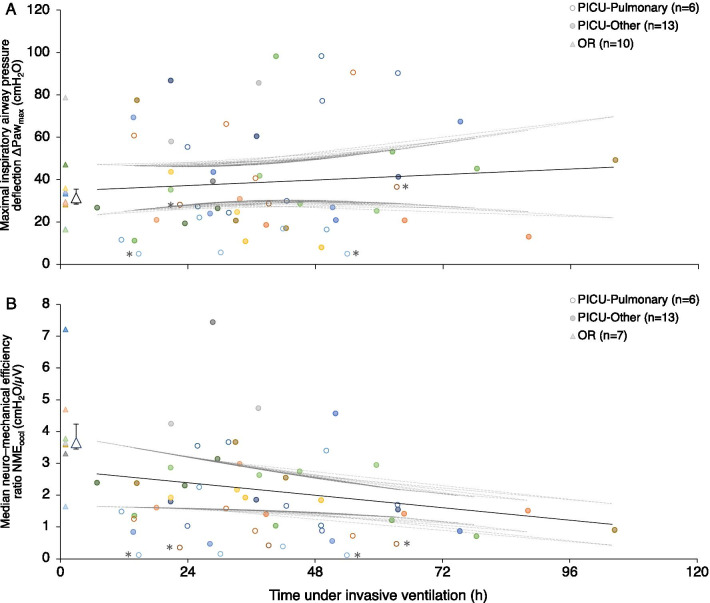
Inspiratory muscle function over time. **A** Maximum inspiratory airway pressure deflection ΔPaw_max_. **B** Median neuro-mechanical efficiency ratio NME_occl_. ΔPaw_max_ denotes maximum inspiratory airway pressure deflection, NME_occl_ median neuro-mechanical efficiency ratio, PICU pediatric intensive care unit (data points marked by empty circles for patients intubated for a lower airway or pulmonary indication and full circles for patients intubated for other indications), OR operating room (data points marked by triangles, group median by large triangle, and first–third quartiles by whiskers). Each color represents an individual patient. The fixed portion of the linear prediction of the mixed model is shown (solid black line) as well as the individual upper and lower 95% confidence intervals (dotted gray lines). *: patients who required non-invasive ventilation continuously for 72 h after extubation

**Fig. 4:**
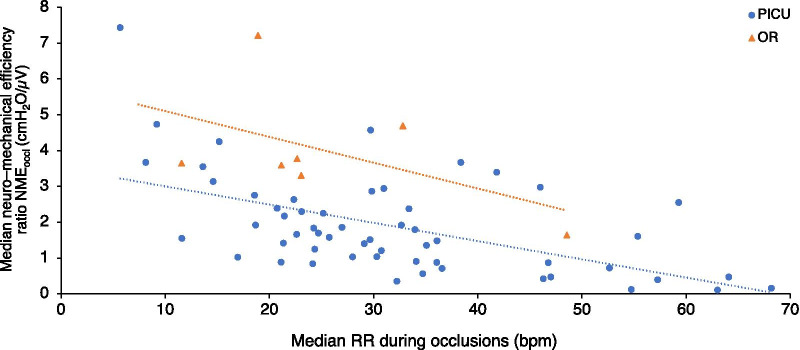
Correlation between the median respiratory rate measured during occlusion maneuvers and the median NME_occl_ for each measurement in both groups. NME_occl_ denotes median neuro-mechanical efficiency ratio, PICU pediatric intensive care unit (data points marked by circles), OR operating room (data points marked by triangles). Dotted lines of best fit according to method of least squares

The evolution of inspiratory muscle strength (ΔPaw_max_) for patients in the PICU group is shown in Fig. 3A. There was no significant change in maximum ΔPaw_max_ over time on MV, with a regression coefficient of 0.109 (95% confidence interval − 0.21 to 0.43, *p* = 0.50). Of the 8 patients (42%) in the PICU group who were not capable of generating a maximum ΔPaw_max_ ≥ 30 cmH_2_O on their last measurement, only 3 could do so on their earliest measurement. The evolution of inspiratory muscles efficiency (NME_occl_) for patients in the PICU group is shown in Fig. 3B. There was a small but significant decrease in median NME_occl_ over time on MV, with a regression coefficient of − 0.016 (95% confidence interval − 0.031 to − 0.002, *p* = 0.03). The evolution of inspiratory muscle function displayed similar patterns in patients intubated for a lower airway or pulmonary indication versus those intubated for other indications (Fig. 3). Of note, no association was observed between the respiratory rate or the EAdi recorded before measurements and the evolution of inspiratory muscle efficiency (data not shown). However, there was a significant negative correlation (*r* = − 0.55 in the PICU group and *r* = − 0.51 in the OR group) between the median frequency of respiratory efforts during occlusion maneuvers and the median NME_occl_ (Fig. 4) for each measurement.

None of the 19 patients required reintubation within 24 h of extubation. Following extubation, 7 patients required NIV for ≤ 24 h, 1 patient required NIV for 32 h, and 2 patients required NIV for > 72 h. Interestingly, these 2 patients had the lowest median NME_occl_ values of all patients (Fig. 3B).

### Comparison between OR and PICU groups (objective 2)

Figure 3 also depicts individual and group values of maximum ΔPaw_max_ (panel A) and median NME_occl_ in the OR group (panel B). Maximum ΔPaw_max_ for the first measurement of the PICU group (35.1 cmH_2_O, *Q*_1_–*Q*_3_ 21–58) and the only measurement of the OR group (31.3 cmH_2_O, *Q*_1_–*Q*_3_ 28.5–35.5) were not statistically significantly different, *p* = 0.982. On the other hand, median NME_occl_ in the PICU group (1.8 cmH_2_O/µV, *Q*_1_–*Q*_3_ 1.3–2.4) was lower than in the OR group (3.7 cmH_2_O/µV, *Q*_1_–*Q*_3_ 3.5–4.2), *p* = 0.015.

## Discussion

The main findings of the present study can be summarized as follows: (1a) recording ΔPaw_max_ and computing a NME_occl_ ratio during an occlusion maneuver is a simple and reliable functional assessment of inspiratory muscles at the bedside of mechanically ventilated children; (1b) inspiratory muscle efficiency (NME_occl_) decreased slightly over time during MV in a group of critically ill children with preserved respiratory drive; (2) inspiratory muscle efficiency (NME_occl_) in critically ill children after 21 h of MV was significantly lower than in children undergoing elective surgery.

### Assessment of inspiratory muscle function

Paw is easy to measure in intubated patients and is directly correlated to esophageal pressure in children [39]. Magnetic stimulation has been used successfully by one group mainly [39–43]; however, our own experience (unpublished observations) is that obtaining reliable diaphragmatic contraction following bilateral anterior magnetic stimulation of the phrenic nerves is challenging in small children. Simply measuring ΔPaw_max_ during a spontaneous breath can be done at the bedside with little additional equipment. However, whereas ΔPaw_twitch_ reflects the function of the diaphragm specifically recruited by phrenic nerve magnetic stimulation, ΔPaw_max_ results from the activity of all inspiratory muscles. ΔPaw_max_ measurements require spontaneous breathing and maximal patient effort. Although this may represent a challenge when performed early in critical illness, including an assessment of respiratory drive results in a useful pre-extubation test [44]. In our PICU group, median ΔPaw increased progressively during an occlusion over the first four breaths but not on the last breath, which would suggest that inspiratory effort was maximal; this was not the case in our OR group, however, as median ΔPaw continuously increased until the last measured breath (Fig. 2A). Other authors have used a unidirectional valve that, unlike the occlusion valve used in this study, allows expiration but not inspiration [45, 46]. This results in larger values of ΔPaw because inspiration occurs from progressively smaller lung volumes and a gradually higher drive. Harikumar et al. [46] suggested that occlusions should be maintained for 12 s or 8 breaths in order to be maximal. We opted to aim for 5 consecutive breathing efforts against occlusion, to be minimally disruptive to patient condition and comfort, as commonly done [44, 45]. We have shown that selecting the maximum value of ΔPaw over up to 5 breaths was the least variable method (Table 5), corresponding to the maximum negative pressure the inspiratory muscles are capable of generating at that time.

By recording EAdi continuously throughout the occlusion maneuvers, we were also able to compute a NME ratio for each breath. The major advantage of using NME alongside ΔPaw resides in how it normalizes pressures generated to respiratory drive by using EAdi. The strength of the diaphragm and other inspiratory muscles can therefore be estimated whether or not the inspiratory effort is maximal, solving many of the issues raised previously. It has also been shown that the NME in any specific patient does not depend on ventilation mode or level of assistance, and that the NME value derived during an expiratory occlusion in the absence of flow is a good surrogate of the NME value measured during regular tidal ventilation [47]. As can be seen in Fig. 3, NME_occl_ values seem more stable to varying patient conditions than ΔPaw_max_ values.

Several limitations of using NME warrant discussion. First, EAdi measurement requires a dedicated catheter and monitoring device, both of which are associated with costs. Any technical difficulties in measuring the EAdi will result in a falsely low EAdi value and consequently an incorrectly high NME ratio. In this study, appropriate catheter positioning was therefore systematically confirmed before each recording using the dedicated software provided [38]. Furthermore, the relationship between ΔPaw and EAdi_max_ may not be linear at very high breathing efforts. In Fig. 2, it is apparent that an increase in EAdi_max_ between the fourth and the fifth breath does not result in a corresponding increase in ΔPaw, likely because the inspiratory muscles have reached maximum pressure-generating capacity that cannot be increased with additional respiratory drive, or because the diaphragm is unable to reach its fully relaxed configuration between breaths. As previously described by others, EAdi values during a specific recording can exhibit breath-to-breath variability (Fig. 1) [48]. Selecting the median value of NME over 5 breaths allowed to reduce this variability below any of the metrics using ΔPaw (Table 5). Finally, whereas EAdi_max_ only measures diaphragm activity, ΔPaw_max_ includes the pressure generated by the diaphragm as well as accessory muscles.

### Evolution of inspiratory muscle function over time

In pediatrics, diaphragm atrophy on MV was only recently demonstrated in studies using ultrasound [13–18]. Although ultrasound overcomes some of the limitations of other imaging modalities such as radiography and fluoroscopy, it provides no information about actual force production. In the current study, which is—to the best of our knowledge—the first to longitudinally assess inspiratory muscle function in children on MV, a small decrease over time in pressure-generating capacity normalized to respiratory drive was found over the whole cohort. These findings taken together suggest that inspiratory muscle atrophy is also associated with a loss in inspiratory muscle efficiency, but the magnitude of the decrease observed was much smaller than that reported in adults [9]. There are various potential reasons why a larger decrease in inspiratory muscle efficiency has not been observed. Because of time required to screen and enroll patients, the first measurement occurred at a median of 20.7 h of MV. Animal data suggest that contractile dysfunction can occur after only 6–12 h of MV [49, 50] and studies in adult ICU patients show that ΔPaw_twitch_ decreases early and in a logarithmic fashion [8]. It is possible that higher NME_occl_ values would have been obtained with earlier initial measurements, resulting in a larger decrease over time. Our measurements were also limited to 72 h after intubation in most patients, but it is possible that a further decrease in efficiency would have been observed on measurements beyond that time in patients with prolonged critical illness and MV. If we consider that ICU–DD comprises two concomitant processes, one related to critical illness and its therapies, and the other related specifically to ventilation (VIDD), their respective impact on inspiratory muscle function may be in opposite directions. The cumulative negative impact of MV could be masked by progressive recovery from critical illness. Lastly, VIDD may not have been significant in our cohort because of a relatively preserved respiratory drive, which also tended to increase over time (Table 4). Low respiratory drive has been shown to be correlated with diaphragmatic atrophy [14]. We have previously reported that respiratory drive, as assessed by EAdi, was frequently blunted on conventional modes of ventilation [35]. EAdi values recorded in the current study were higher than observed in the past. This may be the consequence of a change in clinical practice, potentially compounded by the fact that clinicians were not blinded to the EAdi monitoring and may have aimed to preserve patients’ respiratory drive or transition them to assisted modes of ventilation.

### Clinical implications

At present, there is no recognized definition of ICU–DD in critically ill children. Definitions based on ΔPaw_twitch_ obtained after maximal magnetic stimulation have been proposed in adults [10, 20, 21, 25, 36], but this method is challenging in infants and thus most pediatric studies of diaphragm strength are based on variables comparable to ΔPaw_max_ described here. In a recent study aiming to define normal value ranges in non-intubated healthy children aged 6–11 years old, maximum inspiratory pressure was 85 ± 30 cmH_2_O [51]. In 22 infants and children, ΔPaw_max_ was 31 cmH_2_O after 4.4 days of MV [46]. Khemani et al. recently demonstrated the prognostic significance of ΔPaw_max_ in the PICU: children with ΔPaw_max_ ≤ 30 cmH_2_O at the time of extubation being more likely to be reintubated than those with preserved strength [44]. We report relatively similar ΔPaw_max_ values in our series: from 35 cmH_2_O (Q1–Q3 21–58) on the first measurement to 47.2 cmH_2_O (Q1–Q3 22.2–73.1) on the last measurement. No patient failed extubation in our study and thus we cannot confirm the prognostic value of ΔPaw_max_.

However, our results provide new information by confirming the feasibility of using NME_occl_ to dynamically assess the evolution of diaphragmatic efficiency in critically ill children over time. Several studies have recently reported values for NME in adult ICU patients [52–55]. In patients on conventional ventilation for ≥ 24 h, NME during a spontaneous breathing trial was significantly higher in patients successfully extubated (1.5 cmH_2_O/µV) compared with those who failed extubation (1 cmH_2_O/µV) [52]. In patients who were transitioned from controlled to assisted breathing, NME was 1 cmH_2_O/µV, but did not fluctuate over time and was not correlated with outcomes [53]. In patients on conventional ventilation for ≥ 72 h, NME increased from 1 to 2.6 cmH_2_O/µV after transition to NAVA [54]. Finally, in patients on NAVA for 10 h, NME was 1.2 cmH_2_O/µV and did not change over time [55]. It is important to note that pressure values used to compute NME in these adult studies were extracted directly from the ventilator, not measured through an occlusion valve and manometer, and were therefore impacted by circuit compliance.

There is—to the best of our knowledge—no study reporting NME values in children on conventional ventilation. Wolf et al. have used the related neuro-ventilatory efficiency ratio (NVE, tidal volume/EAdi) to predict successful extubation in PICU patients during a spontaneous breathing trial [56]. In their study, patients who passed an extubation readiness test on pressure support ventilation had a lower NVE than those who failed. Unlike NME, NVE is sensitive to changes in respiratory load (i.e., airway compliance and resistance) and to the work done by the ventilator.

As mentioned above, measuring NME_occl_ (efficiency) provides valuable complementary information to ΔPaw_max_ (strength), which may be of particular importance for longitudinal assessment of inspiratory muscle function by accounting for respiratory drive. Our study design and sample size did not, however, allow for evaluation of the prognostic value of NME in critically ill children. As no patient was re-intubated, we can only note that the only 2 patients in our PICU group who required continuous NIV for 72 h after extubation were also the only patients who had NME_occl_ values < 0.5 cmH_2_O/µV at all timepoints. Although we observed lower neuro-mechanical efficiency (as measured by NME_occl_) in the PICU group, these patients were able to generate similar inspiratory pressures (as measured by ΔPaw_max_) than those in the OR group and none failed extubation. We may speculate that these children were able to compensate for inspiratory muscle weakness by an increase in respiratory drive. More studies are therefore warranted to assess the prognostic value of NME on outcomes such as extubation failure or ventilation duration.

When comparing critically ill children with children undergoing elective surgery, we report NME_occl_ in the PICU group significantly lower than in the OR group. This suggests that inspiratory muscle efficiency seems to be affected rapidly following PICU admission, and therefore likely results from critical illness rather than its therapies. This finding is in line with data observed in adults [10]. Comparison between these two groups must, however, be interpreted with caution. The PICU group included patients that were younger, more patients born prematurely, and some patients who were recently intubated before the current episode. Age was not associated with the level of EAdi in a previous study conducted in similar population [35], but the potential impact of age on NME_occl_ is not known. In terms of medication, only patients in the OR group received sevoflurane, but data suggest it does not have a significant effect on isometric skeletal muscle strength in humans at clinically relevant concentrations [57]. A notable difference in procedure was that occlusion maneuvers were performed without PEEP in the OR group for practical reasons (i.e., interruptions in OR procedural flow). ΔPaw_max_ may vary with lung volume as a function of the force–length relationship of the diaphragm and the varying contribution of passive elastic recoil pressure of the respiratory system [58]. It is theoretically possible that these factors favored higher ΔPaw_max_ (and therefore higher NME_occl_) values in the OR group due to lower initial lung volumes resulting from the absence of PEEP. Most importantly, even if we had initially planned to perform measurements rapidly after intubation in both groups to assess the impact of critical illness without a superimposed difference in time on MV, logistical constraints (e.g., out-of-hours intubation, research personnel availability, etc.) resulted in first measurements being performed only after a median 20.7 h of MV in the PICU group compared to minutes in the OR group.

### Strengths and limitations

The strength of our work resides in the number of breaths analyzed at each time point, pressures measured directly at the endotracheal tube, and a rigorous protocol to maximize EAdi measurement validity. However, our PICU cohort was small, from a single center, and displayed significant heterogeneity in terms of baseline characteristics, indications for PICU admission and intubation, and modes of ventilation. Although this heterogeneity may have decreased the power of this study to detect changes over time, it also reflects the usual PICU population. This would favor clinical relevance and increase the external validity of our findings, together with the fact that measurements were successfully performed in two very different populations (PICU and OR groups). Because our sample did not include many patients with low respiratory drive, it is unclear how applicable these findings would be in such situations.

## Conclusions

In this study, we have proposed and validated an innovative method to reliably measure inspiratory muscle function at the bedside of mechanically ventilated children on a sample aged 1 week to 10 years old. The efficiency of these muscles decreased slightly over time during MV in this PICU cohort with preserved respiratory drive, but was already low on the first measurement after intubation. Further research is needed to explore the relative roles that critical illness (ICU–DD) and MV (VIDD) play in this process and to assess the clinical utility of such monitoring in the PICU setting. This work nevertheless supports the emerging paradigm of aiming for lung-protective but also diaphragm-protective MV [59, 60].

## Data Availability

The datasets used and/or analyzed during the current study are available from the corresponding author on reasonable request, providing approval by the ethics committee of the CHU Sainte-Justine Research Center.

## Abbreviations

CoV: Coefficient of variation
EAdi: Electrical activity of the diaphragm
FiO_2_: Fraction of inspired oxygen
ICU: Intensive care unit
*Q*_1_–*Q*_3_: First and third quartiles
MV: Mechanical ventilation
NAVA: Neurally adjusted ventilatory assist
NIV: Non-invasive ventilation
NME: Neuro-mechanical efficiency ratio
NVE: Neuro-ventilatory efficiency ratio
OR: Operating room
PCO_2_: Partial pressure of carbon dioxide
Paw: Airway pressure
PEEP: Positive end-expiratory pressure
PELOD: Pediatric logistic organ dysfunction
PICU: Pediatric intensive care unit
PSV: Pressure-support ventilation
SpO_2_: Oxygen saturation by pulse oximetry
